# Invasive *Argemone mexicana’s* suppressive effects on germination and early growth of *Triticum aestivum* and *Hordeum vulgare* in South-western Saudi Arabia

**DOI:** 10.1371/journal.pone.0344281

**Published:** 2026-03-06

**Authors:** Manal A. Alshaqhaa, Imen Souid, Manar D. Alshehri, Norah Alyahya, Kamel Msaada, Mounira Mkaddem Guedri

**Affiliations:** 1 Biology Department, College of Science, King Khalid University [KKU], Abha, Saudi Arabia; 2 Aromatic and Medicinal Plants, Biotechnology Center in Borj Cedria Technopole, Hammam-Lif, Tunisia; 3 Laboratory of Energy, Water, Environment and Process (LR18ES35), National Engineering School of Gabes, University of Gabes, Gabes, Tunisia; Arish university, Faculty of agricultural and environmental sciences, EGYPT

## Abstract

The invasion of exotic plant species has emerged as a global problem that impacts the ecosystems, economy, and human health, and is the reason for biodiversity loss. *Argemone mexicana* L. is one of the plants that was recorded as an invasive plant species in south-western Saudi Arabia. Allelochemical properties have been stated but not empirically evaluated on economically important staple crops. In the present study, the phenotype of the *A. mexicana* L. plant was described using major and minor phenotypic morphology, and morphological seed. Additionally, laboratory experiments were conducted to evaluate the allelopathic effects of water extract of *A. mexicana* L. on radicle and plumule length of *Triticum aestivum* and *Hordeum vulgare*. Results showed that the allelopathic potential of leaf and seed extracts of *A. mexicana* decreased the seed germination (until 66.66%), plumule length (93.94%−94.94%), and radicle length (96.68%− 96.96%) respectively for *T. aestivum* and *H. vulgare* with a rise in extract concentration. Moreover, it was observed that the *A. mexicana* seed extract is more allelopathically effective than leaf extract. Hence, it could be concluded that the seed and leaf aqueous extracts contain water-soluble allelochemicals, which could inhibit seed germination of *T. aestivum* and *H. vulgare*.

## I. Introduction

Allelopathy refers to the chemical interactions between plants, whereby certain species release phytochemicals into the environment that can inhibit or stimulate the growth, survival, and reproduction of neighboring plants [1]. These phytochemicals also known as allelochemicals, are synthesized by the vegetation as the secondary products in various biochemical activities [2] and are released either through leaching, exudation, volatilization, decomposition or in leachate forms [3,4]. This process is a significant mechanism for the invasion of alien plants [5]. Invasive alien plants utilize a wide variety of trait strategies, i.e., superior resource use efficiency [6], rapid growth [7,8], high degree of plasticity [9], allelopathy [10,11], and better reproductive performance [12], in the novel environment, which assist them to outcompete the native species, triggering biodiversity loss and ecosystem imbalance [13–15]. *A. mexicana*, for instance, damages native plant species through allelopathy [16], and many common invasive exotic species utilize allelochemicals to exert similar effects [17].

An invasive species, introduced as alien, exotic, and non-native to a location, are often highly competitive and may cause measurable ecological and economic harm.

Globally, invasive alien plants are recognized as major threats to biodiversity con-servation [18], posing threats to both agricultural and natural systems and affect the soil and nutrient cycling of the related ecosystem [19,20]. Many invasive spe-cies are not dominant competitors in their natural habitats but exclude their new neigh-bors in new environments [21,22]. For example, in Saudi Arabia, species such as *Prosopis juliflora* and *Calotropis procera* have been associated with reductions in native vegetation cover, soil degradation, and declines in rangeland productivity [23,24]. Such impacts illustrate the scale at which invasive plants can threaten biodiversity, agriculture, and ecosystem services. Moreover, In Southwestern Saudi Arabia, 48 alien species have been recorded, some of which have been established for decades. *A. mexicana* was one of the documented exotic invasive species exhibiting a medium allelopathic effect on native plants [25]. Southwestern Saudi Arabia is a particularly relevant region for allelopathic studies as it supports both rich native biodiversity and extensive cultivation of staple cereals, particularly wheat (*T. aestivum*) and barley (*H. vulgare*), which are vital for food security. Arid and semi-arid ecosystems are especially vulnerable to biological invasions because low resource availability can magnify competitive effects of invaders. Notwithstanding the fact that harsh environment and climate change nega-tively affect biodiversity, including species like *A. mexicana* [25], taxonomic studies are cru-cial for the effective management of these invasive plants [26].

*A. mexicana* L. (Papaveraceae), which is commonly referred to as Prickly Poppy in English and Premathandu in Tamil, is indigenous to Mexico and has since extensively naturalized in the United States, India, and Ethiopia [27]. A. Mexicana is a widely distrib-uted plant throughout the tropical and subtropical regions of the world. It is found in most places by roadsides and agricultural fields, and it has been recorded for the first time in South-western of Saudi Arabia [25]. Scientific studies have shown that *A. mexicana* contains numerous phytochemicals in high levels, such as carotenoids, phenolic, alkaloids, pec-tins, tannins, coumarins, flavonoids, and terpenoids [28]. Among these, phenolics, alkaloids, and flavonoids are well documented for their allelopathic activity in other invasive species [29,30], suggesting that similar compounds in *A. mexicana* could contribute to its suppressive effects.

Although allelopathic properties of several invasive plants have been reported, empirical data on their effects on economically important cereals in Saudi Arabia are scarce. Previous studies elsewhere have demonstrated that cereals such as rice, wheat, and barley are highly sensitive to allelopathic interactions [31,32]. However, no study has systematically evaluated how *A. mexicana* affects staple crops under Saudi Arabian conditions. Therefore, the present study addresses two central research questions: (i) What are the key morphological characteristics of *A. mexicana* populations in Southwestern Saudi Arabia? and (ii) Do aqueous extracts from the leaves and seeds of *A. mexicana* differentially affect the germination and early seedling growth of *T. aestivum* and *H. vulgare*. It is hypothesized that aqueous extracts of *A. mexicana* leaves and seeds contain biologically active, water-soluble allelochemicals capable of suppressing seed germination and inhibiting early seedling growth (radicle and plumule elongation) in *T. aestivum* and *H. vulgare*. Furthermore, it is expected that seed extracts will exert a greater inhibitory effect than leaf extracts due to higher concentrations or activity of allelopathic compounds.

## II. Materials and methods

Young plant leaves and seeds of *A. mexicana* were collected from different individuals at distinct developmental stages (i.e., young plants for leaves and mature plants for seeds) growing at Al-Soda farms, in South-western region of Saudi Arabia. No ethics committee approval was required for this study, as it did not involve procedures subject to ethical clearance under local regulations; the research was conducted in line with accepted scientific standards and with respect for participants and communities

### 1. Studying the phenotype of plants

Phenotypic traits were evaluated following the standard descriptors recommended by Bioversity International [33]. Ten plants were randomly selected from each accession, and five fully expanded leaves per plant were examined for morphological traits. For seed-related characteristics, thirty seeds per plant were analyzed.

1. The phenotypic morphology of the plant was examined for the root, stem, and leaves in terms of the shape, edge, base, and apex of the blade.
2. The circumferences of Calyx, Corolla, Androecium stamens, and Gynoecium pistils were examined. The morphological features of the fresh parts of *Argemone mexicana* were macroscopically observed and described using sensory organs and using a calibrated ruler.
3. The stomata and trichomes on the leaf surface were examined using Compound Light Microscope. The types of stomata were determined based on models of auxiliary cell arrangement [29], whereas the types of trichomes were determined according to Prabhakar, 2022 [30].
4. Seeds examination: the phenotypic characteristics of the plant’s seed coat were inves-tigated. Moreover, the shape, color, and size of the seeds were determined using Com-pound Light Microscope [31].

### 2. Preparation of the aqueous extract solution

The freshly collected leaves and seeds were washed several times with water, and shade dried at room temperature (25°C – 27°C) for 15 days. Seeds and leaves were separately crushed in a blender into a fine powder. To obtain the extract from leaves or seeds, 500 g refers to dry weight, measured after reaching constant weight, and crushed were soaked separately in a corked, conical containing 1000 mL of distilled water for 72 hours at room temperature with gently agitation and then filtered through Whatman filter paper N° 1. The extracts were diluted to obtain the concentrations of 5g. mL ⁻ ¹ (5%),25 g. mL ⁻ ¹ (25%), 50g.mL ⁻ ¹ (50%), 75g.mL ⁻ ¹ (75%) while the distilled water was used in the control treatment.

### 3. Preparation of the crops seeds and the experiment design

**Fig 1:**
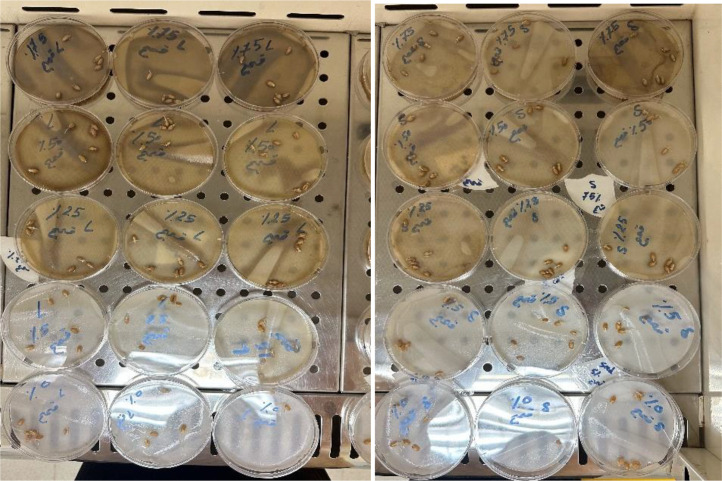
The experimental design for the treatment of *Triticum aestivum* using different concentrations of seeds and leaves aqueous extracts (5, 25, 50, and 75 g·mL ⁻ ¹) obtained from *Argemone mexicana* plant.

**Fig 2:**
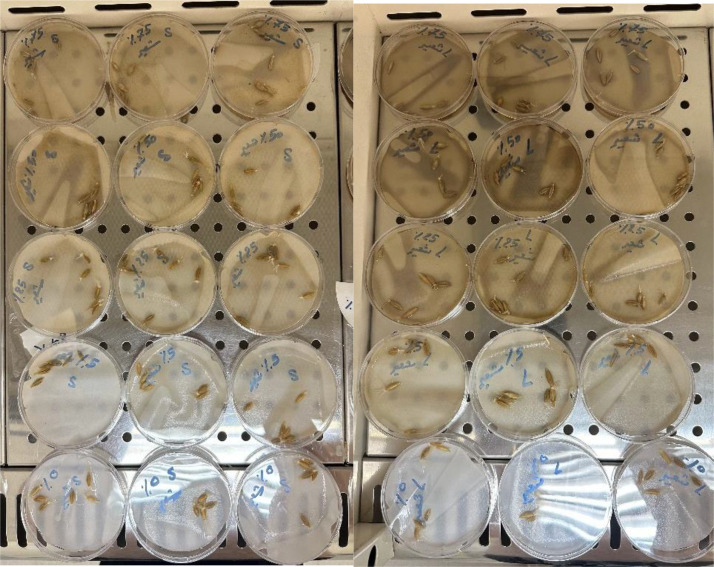
The experimental design for the treatment of *Hordeum vulgare* using different concentrations of seeds and leaves aqueous extracts (5, 25, 50, and 75 g·mL ⁻ ¹) obtained from *Argemone mexicana* plant.

The seeds of *T. aestivum* and *H. vulgare* were procured from the Agricultural Office. The seeds” of *T. aestivum* and *H. vulgare* were surface sterilized with 0.1% mercuric chloride for 1 min to eliminate the fungal spores on the seeds (Future investigations will employ safer alternative). Then the seeds were washed with distilled water many times to remove the mercuric chloride. The seeds were soaked in different concentrations of *A. mexicana* extracts for 24 hours and then placed in 9 cm Petri dishes lined with sterile cotton. Each Petri-dish contained 6 normal-sized seeds (based on average diameter and weight), with three Petri dishes (replicates) prepared for every treatment concentration (i.e., a total of 18 seeds per concentration) which were irrigated with 20 ml distilled water on alternative days. Seeds soaked with distilled water were maintained as control separately. The experiment followed a randomized complete block design (RCBD) with 3 blocks (replications) and 5 treatments randomized within each block. The petri dishes were sealed with caps and kept inside the cupboards at room temperature (22–25 °C) (Fig 1 and 2).

Extract/distilled water (a standardized volume of 5 ml) was added to moisten the seeds when required. Seeds were observed every day. Petri dish is considered the experimental unit and the germinated seed is defined consistently (e.g., radicle ≥ 2 mm).

During the nine days of treatment, plantlets were used for the measurement of the radicle and plumule lengths. The individual radicles/plumules length values were then noted.

### 4. Data collection and calculations

Seed germination percentage, germination speed, mean germination time and mean daily germination were determined following formula described by (Damalas et al. 2019) [34]. Germination index was determined following formula described by (Akbar et al. 2025) [35]. Germination potential was calculated according to Liu et al., 2015.


$$\text{Germination }\%=\frac{\text{Number of germinated seeds in each Petri dish}}{\text{Total number of seeds in Petri dish}}\;\times \text{ 100}$$ (1)

$$\text{Germination speed}=\frac{n\text{1}}{d\text{1}}+\frac{\text{n2}}{d\text{2}}+\frac{\text{n3}}{d\text{3}}\pm \ldots$$ (2)

Where n1 = number of seeds germinated per Petri dish on day d1,

n2 = number of seeds germinated on Petri dish on day d2,

n3 = number of seeds germinated on Petri dish on day d3.


$$\text{Mean germination time}=\frac{\sum \text{n}.\text{d}}{\sum \text{n}}\;$$ (3)

where n = number of seeds newly germinated at time d in each Petri dish,

d = days from the beginning of the germination test,

∑n = number of seeds at the final germination.


$$\text{Mean daily germination}=\frac{\text{Total number of seeds germinated in a Petri dish}}{\text{Total number of days}}$$ (4)

$$\text{Germination index}=\;\frac{Number\;of\;seeds\;germinated\;in\;a\;Petri\;dish\;on\;day\;X}{Day\;X}+\ldots \;+\frac{umber\;of\;seeds\;germinated\;in\;a\;Petri\;dish\;on\;day\;Y}{Day\;Y}$$ (5)

Where the sum (day X + … + day Y) includes all days of observation.


$$\text{Germination potential}=\;\frac{Number\;of\;seeds\;germinated\;by\;day\;X\;in\;each\;Petri\;dish\;}{Total\;number\;of\;seeds\;in\;Petri\;dish}\;x\;\text{1}\text{0}\text{0}$$ (6)

where X = 6

Each treatment of this experiment was conducted with 3 replications and repeated twice.

### 5. Statistical analysis

To assess difference in germination and the elongation data among different treatments, we conducted two-way independent (concentration × plant part) analysis of variance (ANOVA). Prior to the ANOVA, the normality was examined by using Shaprio-Wilks test. Also, the variance homogeneity test (Leven’s test) for each group was performed and data were transformed as necessary. Duncun’s test was used to determine differences between means at p < 0.05. All data were presented as mean ± standard error (SE). A simple linear regression model was employed to investigate the effects of the *A. mexicana* exrtracts on the germination indexes and the plumule and radicle elongation at significance level of 0.05, and a desired power of 95%. We used IBM SPSS statistics (version 23.0, 2015, Armonk, NY, USA) to conduct all analyses.

## III. RESULTS

### 3.1. Phenotypical characteristics of the vegetative and flowering stages

#### 3.1.1. Biological and growth nature.

**Fig 3:**
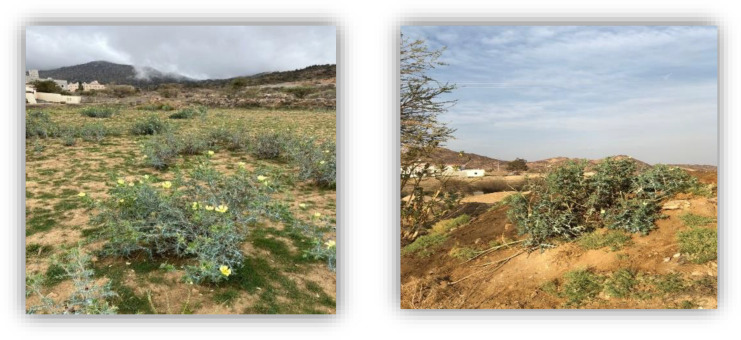
Natural habitat and morphological features of *Argemone mexicana* L. growing in the farmlands of Al-Soda village, Abha, Saudi Arabia.

*A. mexicana* is an upright herbaceous annual plant, ranging in height from 60 to 110 cm, spreads on roadsides, ravine sides and in agricultural areas. This study documented its presence in south-western Saudi Arabia, between latitudes 17°25’ N and 19°50’ N and longitudes 41°50’ E and 44°00’ E. Its presence was observed in the Farmlands of Abha, Al-Soda, Al-Mashhad and Al-Faraa cities and villages (Fig 3).

#### 3.1.2. Morphological characteristics.

*A. mexicana* is an annual herb up to 150 cm long with a slightly branched peg root. The plant is upright, branched, usually prickly, pale bluish-green, and exuding foul-smelling yellow sap when cut. The research findings showed that the aerial parts of *A. mexicana* ranged from 90 cm to approximately 110 cm.

The stem is herbaceous and slightly branched, the vegetative part is spreading and expanding, its diameter (the canopy width of the plant) reaches approximately 1 m, the stem appears cylindrical in cross-section.

The leaf is lobed, petiolate, with spiny edges, and the edge is serrated. It alternates on the branch. The leaves are gray-green in color, thick and leathery. The blade is cleft into rounded segments, pinnate, 6 to 15 cm long, and 3 to 8 cm wide. The veining is feathery reticulate, white in color, and very distinct.

**Fig 4:**
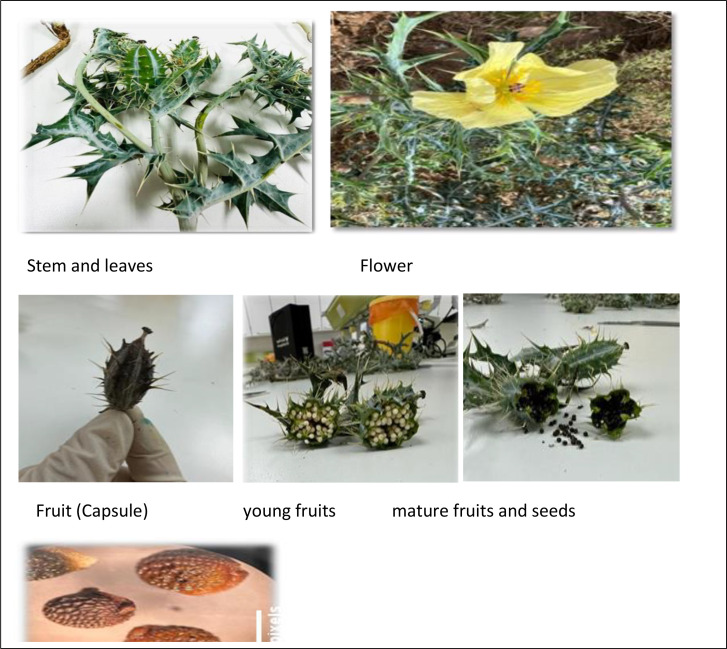
Vegetative and reproductive organs of *Argemone mexicana*: stem and leaf, flower, fruit, and seed.

The flowers are six-parted, complete, containing the female and male reproductive organs and actinomorphic in symmetry. The flower buds are spherical. The calyx consists of 3 separate green sepals that are oval, and the corolla has 4–6 bright yellow rounded petals, 4 cm long and 3 cm wide (flowers 4 to 7 cm in diameter). The androecium is long stamens from 5 to 10 stamens 10 mm long, arranged in a spiral, at two diameters. The gynoecium consists of several fused carpels, the ovary is upper, 10 to 15 mm long, with 6 fused carpels (each containing 4–5 rows of ovules), and bears 5 stigmas, taking on a reddish-purple color, appearing on top of the ovary in the flower (Fig 4). Fruit is a prickly capsule, oblong or ovoid; seeds are brown, nearly spherical, 1.7–2 mm in diameter, with reticulate surface (Fig 4).

Leaf surfaces lacked trichomes, but thorns were present throughout, including on the leaf surfaces and along the medial vein. The stomata spread on the two surfaces of the leaf, and it contained two patterns of stomata. The first pattern is Anisocytic auxiliary cells, where the stomata are surrounded by renal-shaped guard cells, while the auxiliary cells have irregular edges and are uneven in size, and their number is three cells.

**Fig 5:**
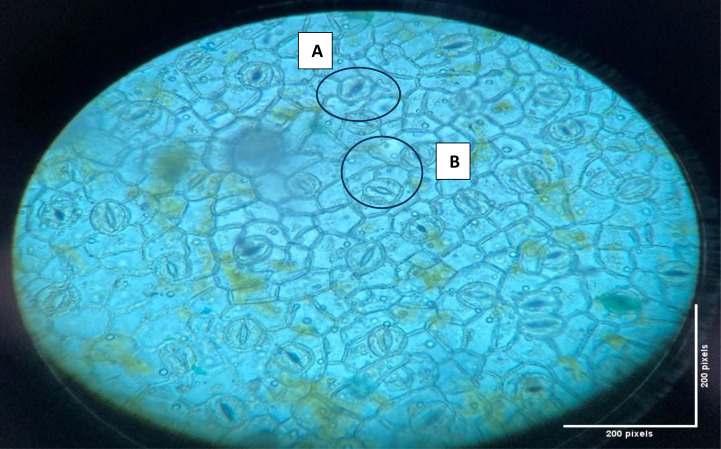
The stomata types of *A. mexicana* leaves, A: Anisocytic; B: Actinocytic.

The second pattern is Actinocytic auxiliary cells which encircle the guard cells in radiating form and are irregular in number (5–7 cells) as depicted in (Fig 5).

The dual stomatal patterns observed could potentially represent an adaptive trait, although further ecophysiological investigations are required to substantiate this hypothesis.

### 3.2. Effects of *A. mexicana* leaf and seed aqueous extracts on *T. aestivum and H. vulgare* germination

The germination of *T. aestivum* and *H. vulgare* under the treatment of *A. mexicana* aqueous extracts was investigated and shown in Table 1. For *T. aestivum*, the interaction between water extract concentration and *A. mexicana* plant part were significant for germination percentage, mean germination speed and germination potential (*p* < 0.05). Although, the individual impact of concentration was found to be significant for all germination variables (*p* < 0.05). Plant part as separate factor had no significant impact on mean daily germination. According to *H. vulgare*, the interaction between *A. mexicana* plant part and extract concentration were not significant for germination percentage, mean germination speed, mean germination time, germination index and germination potential, whereas the interaction was statistically significant for mean daily germination (*p* < 0.05). The concentration as separate factor was significant for all germination variables (*p* < 0.05). *A. mexicana* plant part as a separate factor was significant (*p* < 0.05) for only mean daily germination (Table 1).

**Table 1:** ANOVA output (F-ratios) displaying the effects of plant part (PP) and extract concentrations (EC) on germination parameters of *T. aestivum* and *H. vulgare.*

Parameter	PP	EC	PP x EC
***T. aestivum***			
**Germination (%)**	F_1,80_ = 51.200*	F_4,80_ = 38.000*	F_4,80_ = 5.200*
**Germination speed**	F_1,80_ = 41.083*	F_4,80_ = 38.293*	F_4,80_ = 4.250*
**Mean germination time**	F_1,80_ = 19.769*	F_4,80_ = 23.931*	F_4,80_ = 2.543
**Mean daily germination**	F_1,80_ = 1.160	F_4,80_ = 19.811*	F_4,80_ = 0.174
**Germination index**	F_1,80_ = 13.444*	F_4,80_ = 18.722*	F_4,80_ = 2.056
**Germination potential**	F_1,80_ = 38.293*	F_4,80_ = 41.083*	F_4,80_ = 4.250*
***H. vulgare***			
**Germination (%)**	F_1,80_ = 0.020	F_4,80_ = 9.102*	F_4,80_ = 0.429
**Germination speed**	F_1,80_ = 0.996	F_4,80_ = 5.102*	F_4,80_ = 0.448
**Mean germination time**	F_1,80_ = 0.883	F_4,80_ = 8.685*	F_4,80_ = 0.381
**Mean daily germination**	F_1,80_ = 17.000*	F_4,80_ = 241.235*	F_4,80_ = 10.235*
**Germination index**	F_1,80_ = 0.344	F_4,80_ = 8.896*	F_4,80_ = 0.374
**Germination potential**	F_1,80_ = 1.185	F_4,80_ = 8.583*	F_4,80_ = 0.398

* Significant at *p* < 0.05.

As shown in Tables 2 and 3, the aqueous extracts had noteworthy effect on germination variables of the treated plants in comparison to the control. Increasing the concentrations of both *A. mexicana* leaves and seeds extracts significantly decreased all germination variables. *A. mexicana* extracts exhibited inhibitory effect on germination percentage, mean germination speed, mean germination time, mean daily germination, germination index and germination potential of both *T. aestivum* and *H. vulgare* with stronger inhibition observed at higher concentrations. For instance, the 50% and 75% *A. mexicana* leaves extract concentrations reduced significantly the mean germination percentage of *T. aestivum* plants (83.33%−66.66%), the mean germination speed (2.20–1.89) and the mean daily germination (0.44–0.37). In addition, a significant difference in the mean germination time, the germination index (2.44) and the germination potential (66.66) was observed under leaves extract concentration of 75% (Table 2). However, the seeds extract concentrations of 25%, 50% and 75% reduced considerably the mean germination time. The seeds extract concentrations of 50% and 75% decreased significantly the mean germination speed, the mean daily germination, and the germination potential as well.

**Table 2:** Effect of *A. mexicana* leaf and seed aqueous extracts on seed germination of *T. aestivum.*

Concentration (%)	Plant part	Germination (%)	Germination speed	Mean germination time	Mean daily germination	Germination index	Germination potential
**0**	Leaf	100.00 ± 0.00^a^	3.67 ± 0.39^a^	18.00 ± 0.00^a^	0.67 ± 0.01^a^	3.67 ± 0.00^a^	100.00 ± 0.00^a^
	Seed	100.00 ± 0.00^a^	3.67 ± 0.39^a^	18.00 ± 0.00^a^	0.67 ± 0.01^a^	3.67 ± 0.00^a^	100.00 ± 0.00^a^
**5**	Leaf	94.61 ± 5.55^ab^	3.06 ± 0.22^ab^	17.83 ± 0.16^a^	0.59 ± 0.05^ab^	3.55 ± 0.11^a^	100.00 ± 0.00^a^
	Seed	72.38 ± 5.55^c^	3.02 ± 0.24^ab^	16.83 ± 0.92^ab^	0.58 ± 0.05^ab^	3.02 ± 0.17^abc^	94.44 ± 5.55^a^
**25**	Leaf	89.05 ± 5.55^ab^	2.96 ± 0.29^ab^	16.83 ± 0.92^ab^	0.56 ± 0.03^abc^	3.35 ± 0.18^ab^	94.44 ± 5.55^a^
	Seed	77.77 ± 5.55^bcd^	2.85 ± 0.30^ab^	14.00 ± 1.75^bc^	0.51 ± 0.01^abcd^	2.85 ± 0.25^bc^	77.78 ± 11.11^abc^
**50**	Leaf	83.33 ± 0.00^b^	2.20 ± 0.13^b^	15.83 ± 0.83^ab^	0.44 ± 0.07^bcd^	3.14 ± 0.09^ab^	88.88 ± 5.55^ab^
	Seed	50.00 ± 0.00^d^	2.02 ± 0.17^b^	10.66 ± 0.83^d^	0.38 ± 0.02 cd	2.02 ± 0.09^de^	61.11 ± 5.55^c^
**75**	Leaf	66.66 ± 0.00^c^	1.89 ± 0.13^b^	12.00 ± 0.01 cd	0.37 ± 0.04^d^	2.44 ± 0.00 cd	66.66 ± 0.00^bc^
	Seed	44.44 ± 5.55^d^	1.81 ± 0.15^b^	9.66 ± 0.92^d^	0.34 ± 0.02^d^	1.81 ± 0.17^e^	55.55 ± 5.55^c^

Data represented as mean±SE. Means in the same column followed by different letters are significantly different (*p < 0.05*).

**Table 3:** Effect of *A. mexicana* leaf and seed aqueous extracts on seed germination of *H. vulgare.*

Concentration (%) or (g.mL^-1^)	Plant part	Mean germination (%)	Mean germination speed	Mean germination time	Mean daily germination	Germination index	Germination potential
**0**	Leaf	100.00 ± 0.00^a^	3.67 ± 0.39^a^	18.00 ± 0.00^a^	0.67 ± 0.01^a^	3.66 ± 0.00^a^	100.00 ± 0.00^a^
	Seed	100.00 ± 0.00^a^	3.67 ± 0.39^a^	18.00 ± 0.00^a^	0.67 ± 0.01^a^	3.66 ± 0.00^a^	100.00 ± 0.00^a^
**5**	Leaf	78.10 ± 5.55^ab^	3.10 ± 0.27^ab^	16.50 ± 0.99^ab^	0.59 ± 0.06^b^	3.13 ± 0.20^ab^	94.44 ± 5.55^a^
	Seed	78.10 ± 5.55^ab^	2.85 ± 0.31^ab^	14.00 ± 0.10^abcd^	0.52 ± 0.01^c^	2.85 ± 0.20^ab^	77.78 ± 5.55^ab^
**25**	Leaf	78.10 ± 5.55^ab^	3.04 ± 0.30^ab^	15.66 ± 0.92^abc^	0.56b ± 0.02^c^	3.04 ± 0.17^ab^	88.89 ± 5.55^ab^
	Seed	66.66 ± 16.66^abc^	2.44 ± 0.26^abc^	12.00 ± 3.00^abcde^	0.44 ± 0.01^d^	2.44 ± 0.61^ab^	66.67 ± 16.66^ab^
**50**	Leaf	38.88 ± 14.69^bc^	1.42 ± 0.12^b^	8.66 ± 3.31^cde^	0.31 ± 0.02^ef^	1.61 ± 0.61^ab^	50.00 ± 19.24^ab^
	Seed	55.55 ± 20.02^bc^	2.04 ± 0.22^bcd^	10.00 ± 3.60^bcde^	0.37 ± 0.01^e^	2.04 ± 0.73^ab^	55.55 ± 20.02^ab^
**75**	Leaf	38.88 ± 19.24^bc^	1.43 ± 0.16 cd	7.00 ± 1.99 cd	0.26 ± 0.14^g^	1.43 ± 0.40^ab^	38.89 ± 11.11^ab^
	Seed	27.77 ± 20.02^d^	1.02 ± 0.11^d^	5.00 ± 3.60^d^	0.19 ± 0.01^h^	1.02 ± 0.73^b^	27.78 ± 20.02^b^

Data represented as mean±SE. Means in the same column followed by different letters are significantly different (*p < 0.05*).

On the other hand, the *A. mexicana* leaves extract concentrations of 5%, 25%, 50% and 75% reduced mean daily germination of *H. vulgare*. Moreover, leaves extract concentrations of 50% and 75% reduced significantly mean germination percentage and mean germination speed and germination potential. However, the *A. mexicana* leaves extract had no effect on the mean germination time, germination index and germination potential. *A. mexicana* seeds extract concentrations of 75% decresed remarkably the mean germination percentage, the mean germination speed, mean germination time, germination index and germination potential (Table 3).

### 3.3. Effects of *A. mexicana* leaf and seed aqueous extracts on the plumule and radicle elongation of *T. aestivum*

The effects of *A. mexicana* plant part and the extract concentration as well as their interaction on the *T. aestivum* plumule and radicle elongation were presented in Table 4. However, the shown interaction was not significant for the radicle growth, it significantly influenced the plumule length on the 6^th^ and the 9^th^ days of treatment. Extract concentration as separate factor was significant (*p* < 0.05) for all elongation variables. Plant part as separate factor was significant for the plumule elongation on the 6^th^ and 9^th^ day of treatment (*p* < 0.05). Also, *A. mexicana* plant part significantly affected the radicle length on the 3^rd^ day of treatment (*p* < 0.05).

**Table 4:** Two-way ANOVA output (F-ratios) displaying the effects of plant part (PP), extract concentration (EC) and their interaction (PP x EC) on plumule and radicle elongation of *T. aestivum.*

Parameter	PP	EC	PP x EC
**Plumule length**			
**Day 3**	F_1,80_ = 1.374	F_4,80_ = 13.010*	F_4,80_ = 1.751
**Day 6**	F_1,80_ = 7.024*	F_4,80_ = 75.853*	F_4,80_ = 6.848*
**Day 9**	F_1,80_ = 37.490*	F_4,80_ = 100.883*	F_4,80_ = 11.489*
**Radicle length**			
**Day 3**	F_1,80_ = 6.430*	F_4,80_ = 56.434*	F_4,80_ = 1.165
**Day 6**	F_1,80_ = 0.313	F_4,80_ = 123.888*	F_4,80_ = 0.088
**Day 9**	F_1,80_ = 0.017	F_4,80_ = 212.490*	F_4,80_ = 1.209

*Significant at *p* < 0.05.

**Fig 6:**
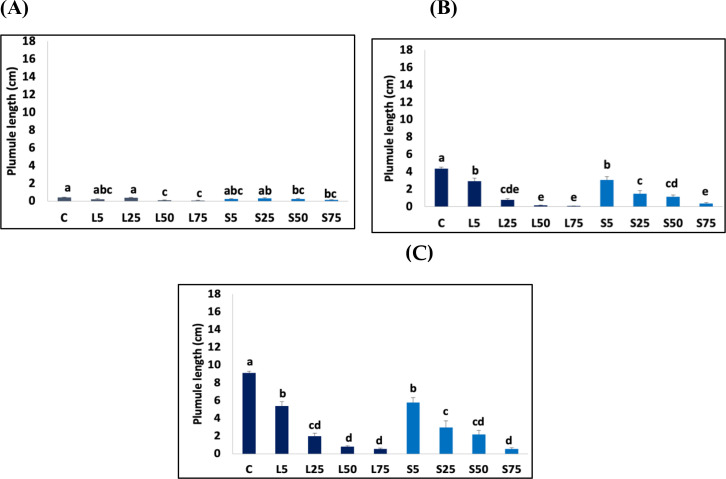
The effect of *Argemone mexicana* leaf and seed extract treatments on Triticum aestivum plumule elongation (cm). **(A)** At 3 days of treatment; **(B)** At 6 days of treatment; **(C)** At 9 days of treatment. C indicates control. L5, L25, L50, and L75 represent aqueous leaf extract concentrations of 5 g·mL ⁻ ¹, 25 g·mL ⁻ ¹, 50 g·mL ⁻ ¹, and 75 g·mL ⁻ ¹, respectively. S5, S25, S50, and S75 represent aqueous seed extract concentrations of 5 g·mL ⁻ ¹, 25 g·mL ⁻ ¹, 50 g·mL ⁻ ¹, and 75 g·mL ⁻ ¹, respectively. Data are represented as mean ± SE. Bars with different letters are significantly different at *p < 0.05.*

In the current investigation, the highest lengths of T*. aestivum* plumules and radicles were observed on the third day of treatment in control plants (0.39 cm and 1.42 cm, respectively). Although the *A. mexicana* leaf and seed extracts had a substantial inhibitory impact on the plumule elongation of *T. aestivum* at the concentration of 50%, higher concentrations of the aqueous extract had no stronger inhibitory effect (Fig 6).

The regression analysis of plumule length and the concentration of leaf and seed extracts showed coefficients of determination (R^2^) of 0.65 and 0.61, respectively. This implies that more than 61% of variation in *T. aestivum* plumule elongation could be explained by the concentration of either leaf or seed extracts after 3 days of treatment.

**Fig 7:**
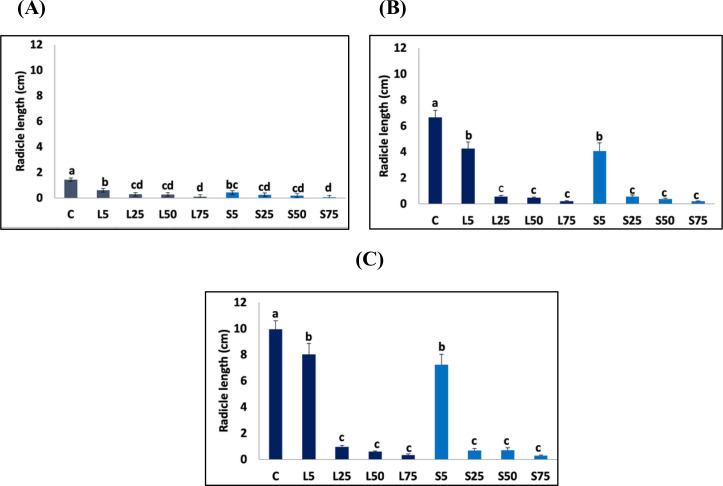
The effect of *A. Mexicana* leaf and seed extracts treatment on *T. aestivum* radicle elongation (cm) (A) At 3 days of treatment (B) at 6 days of treatment (C) At 9 days of treatment. C indicates control, L5, L25, L50 and L75 represent aqueous leaf extract concentrations of the 5 g.mL^-1^, 25 g.mL^-1^, 50 g.mL^-1^ and 75 g.mL^-1^. S5, S25, S50 and S75 represent aqueous seed extract concentrations of the 5 g.mL^-1^, 25 g.mL^-1^, 50 g.mL^-1^ and 75 g.mL^-1^. Data are represented as mean±SE. Bars with different letter (s) are significantly different at *p < 0.05*.

Nevertheless, *T. aestivum* radicles were more sensitive to the *A. mexicana* aqueous extracts on the 3^rd^ day of treatment. In fact, the inhibitory effect of such solutions on the radicle growth was detected at the concentration of 5%, while higher concentrations of aqueous extracts did not exhibit a significantly enhanced inhibitory effect (Fig 7). Fig 7. The effect of *Argemone mexicana* leaf and seed extract treatments on *Triticum aestivum* radicle elongation (cm). (A) At 3 days of treatment; (B) At 6 days of treatment; (C) At 9 days of treatment. C indicates control. L5, L25, L50, and L75 represent aqueous leaf extract concentrations of 5 g·mL ⁻ ¹, 25 g·mL ⁻ ¹, 50 g·mL ⁻ ¹, and 75 g·mL ⁻ ¹, respectively. S5, S25, S50, and S75 represent aqueous seed extract concentrations of 5 g·mL ⁻ ¹, 25 g·mL ⁻ ¹, 50 g·mL ⁻ ¹, and 75 g·mL ⁻ ¹, respectively. Data are represented as mean ± SE. Bars with different letters are significantly different at *p < 0.05.*

The determination coefficient (R^2^) of the radicle length and both leaf and seed extract concentrations was 0.61 and 0.53, respectively.

After 6 days of treatment, a significant decrease in plumule size was observed at the concentration of 5% of leaf and seed extracts*.* This effect was more important at the extract concentration of 75% where the elongation was reduced by 98.63 ± 1.47% under leaf extract and by 91.53 ± 4.80% under seed extract treatments (Fig 6).

The regression analysis established that 77% of variance in plumule elongation of *T. aestivum* may be attributed to the concentration of leaf extracts (R^2^ = 0.77) and 84% variation is due to the concentration of seed extracts (R^2^ = 0.84).

On the 6^th^ day of treatment, the *T. aestivum* radicle exhibited sensitivity to 25% leaf and seed extracts, resulting in a notable reduction in growth (Fig 7).

This inhibitory effect increased at higher concentration of the aqueous extracts. For instance, at leaf or seed extract concentrations of 75%, the *T. aestivum* radicle size showed a reduction of 96.98 ± 0.59% comparing to the control. The regression analysis of radicle length and concentration of leaf extracts showed a coefficient of R^2^ = 0.69, whereas the regression analysis of *T. aestivum* radicle length and the concentration of seed extracts showed a coefficient of R^2^ = 0.68.

After 9 days of treatment, the significant inhibitory effect of the leaf and seed extracts on the *T. aestivum* plumule and radicle was shown at the concentration of 5%*.* At a concentration of 75% of leaf and seed extracts, reduction of 93.94 ± 2.48% and 93.94 ± 3.23%, respectively were noted on the plumule elongation when compared to the control. The regression analysis reported that 74% of this variation was due to leaf extract concentrations (R^2^ = 0.74) and 82% of variability was due to the seed extract concentrations. In addition, at the concentration of 75%, the radicle size of *T. aestivum* exhibited a diminution of 96.68 ± 0.73% when treated with leaf extract and 97.18 ± 0.46% when treated with seed extract. The regression analysis of radicle length and concentration of leaf extract was R^2^ = 0.72, while the regression analysis of radicle length and seed extract concentrations was R^2^ = 0.71(Figs 6 and 7).

### 3.4. Effects of *A. mexicana* leaf and seed aqueous extracts on the plumule and radicle elongation of *H. vulgare*

**Fig 8:**
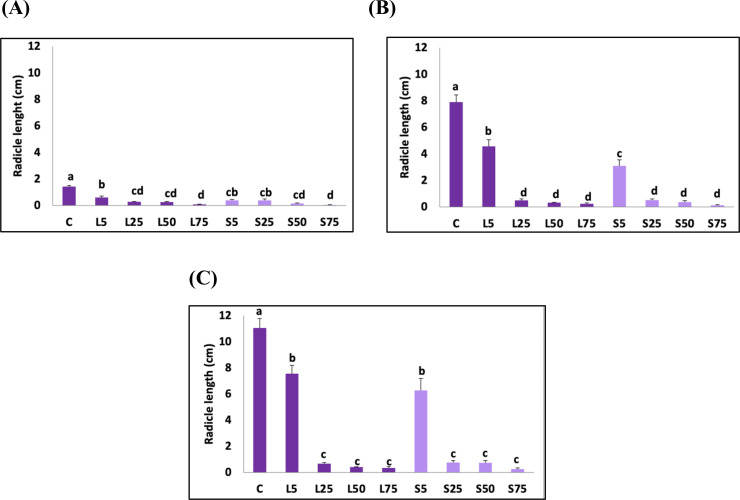
The effect of *A. Mexicana* leaf and seed extracts treatment on *H. vulgare* radicle elongation (cm) (A) at 3 days of treatment (B) at 6 days of treatment (C) at 9 days of treatment. C indicates control, L5, L25, L50 and L75 represent aqueous leaf extract concentrations of the 5 g.mL^-1^, 25 g.mL^-1^, 50 g.mL^-1^ and 75 g.mL^-1^. S5, S25, S50 and S75 represent aqueous seed extract concentrations of the 5 g.mL^-1^, 25 g.mL^-1^, 50 g.mL^-1^ and 75 g.mL^-1^. Data are represented as mean±SE. Bars with different letter (s) are significantly different at *p < 0.05*.

The effects of *A. mexicana* plant part and the extract concentration as well as their interaction on *H. vulgare* plumule and radicle elongation were shown in Table 5 and Fig 8. The interaction between plant part and extract concentration was significant for the plumule length on the 6^th^ and the 9^th^ days of treatment. For the radicle elongation, this interaction was significant on the 6^th^ day, only. Extract concentration as separate factor was significant (*p* < 0.05) for all elongation variables. *A. mexicana* plant part as separate factor was significant for the plumule elongation on the 6^th^ day (*p* < 0.05). Also, plant part significantly affected the radicle length on the 3^rd^ and the 6^th^ day (*p* < 0.05).

**Table 5:** ANOVA output (F-ratios) displaying the effects of plant part (PP), extract concentration (EC) and their interaction (PP x EC) on plumule and radicle elongation of *H. vulgare.*

Parameter	PP	EC	PP x EC
**Plumule length**			
**Day 3**	F_1,80_ = 0.088	F_4,80_ = 10.912*	F_4,80_ = 0.648
**Day 6**	F_1,80_ = 4.988*	F_4,80_ = 71.165*	F_4,80_ = 3.432*
**Day 9**	F_1,80_ = 23.511	F_4,80_ = 687.127*	F_4,80_ = 49.797*
**Radicle length**			
**Day 3**	F_1,80_ = 4.016*	F_4,80_ = 50.714*	F_4,80_ = 1.926
**Day 6**	F_1,80_ = 13.745*	F_4,80_ = 140.316*	F_4,80_ = 5.552*
**Day 9**	F_1,80_ = 0.834	F_4,80_ = 149.214*	F_4,80_ = 0.676

*Significant at *p* < 0.05.

**Fig 9:**
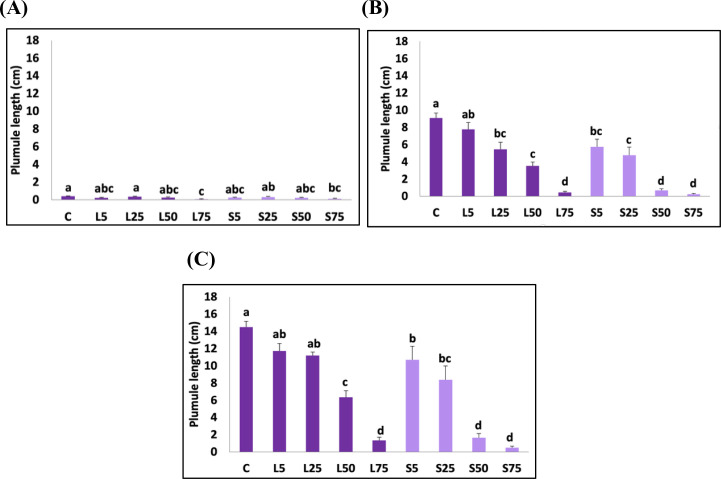
The effect of *A.* *Mexicana* leaf and seed extracts treatment on *H. aestivum* plumule elongation (cm) (A) at 3 days of treatment **(B)** At 6 days of treatment (C) at 9 days of treatment. C indicates control, L5, L25, L50 and L75 represent aqueous leaf extract concentrations of the 5 g.mL^-1^, 25 g.mL^-1^, 50 g.mL^-1^ and 75 g.mL^-1^. S5, S25, S50 and S75 represent aqueous seed extract concentrations of the 5 g.mL^-1^, 25 g.mL^-1^, 50 g.mL^-1^ and 75 g.mL^-1^. Data are represented as mean±SE. Bars with different letter (s) are significantly different at *p < 0.05*.

The allelopathic effect of *A. mexicana* leaf and seed extracts on *H. vulgare* plumule and radicle growth was investigated and presented in Figs 8 and 9.

The obtained results showed that exhibiting the *H. vulgare* plant to the 75% aqueous extracts of *A. mexicana* significantly decreases the plumule growth starting from the 3^rd^ day of treatment. The determination coefficient (R^2^) of the plumule length and concentration of leaf and seed extracts were of 0.56 and 0.76, respectively. However, 5% leaf or seed extracts effectively reduced the radicle elongation with higher concentrations resulting in greater radicle growth inhibition after 3 days of treatment. The coefficient of determination (R^2^) of the radicle length and concentration of leaf and seed extracts were 0.61 and 0.56, respectively.

On the 6^th^ day of treatment, although the significant decrease observed in the plumule size of *H. vulgare* at the concentrations of 25% leaf extract and 5% seed extract, treating *H. vulgare* plants with 75% concentration of leaf and seed extracts significantly reduced the plumule elongation by 94.94 ± 1.54% and 97.36 ± 0.92%, respectively. The regression analysis showed that 99% of variation in plumule length of *H. vulgare* may attribute to the concentration of leaf extract concentrations (R^2^ = 0.99) and seed extract concentrations (R^2^ = 0.88).

Furthermore, *H. vulgare* consistently exhibited sensitivity to the lowest leaf and seed extracts (5%) on the sixth day of treatment, resulting in a considerable decrease in elongation. The shortest radicle was observed in the samples treated with the extracts at 75% concentration. At this concentration, the reduction in radicle size was 96.96 ± 0.92% with leaf extract and 98.36 ± 0.64% with seed extract. The coefficient of determination (R^2^) of the radicle length and concentration of leaf and seed extracts were 0.65 and 0.57, respectively.

On the 9^th^ day of treatment, the inhibitory effect on *H. vulgare* plumule growth was observed at 50% concentration of leaf extract and at 5% of seeds extract. Furthermore, the results revealed that the shortest plumule of *H. vulgare* was 1.32 cm in plants treated with 75% leaf extract which implied a diminution of 90.89 ± 2.84% comparing to the control and 0.5 cm in plants treated with 75% seed extract with a diminution of 96.55 ± 0.93%. The regression analysis of plumule length and concentration of leaf and seed extracts recorded the regression coefficients R^2^ = 0.96 and R^2^ = 0.93, respectively.

The radicle elongation of *H. vulgare* significantly decreased when subjected to 5% leaf and seed extracts over a duration of 9 days, as demonstrated for *T. aestivum* (Fig 9). The radicle size of *H. vulgare* showed a diminution of 96.83 ± 1.24% and 97.74 ± 0.80% when treated with 75% leaf and seed extracts, respectively. The regression analysis of radicle length and concentration of leaf extract showed a variance of 68% (R^2^ = 0.68). The seed extract on the other hand, had 66% variation in radicle length of *H. vulgare* (R^2^ = 0.66).

## IV. Discussion

### 1. Morphological observations

*A. mexicana* has been morphologically examined in the current study and found that the plant possesses a lobed, spiny-edged leaf structure with a pinnate blade and dis-tinct white venation-a trait characteristic of the species. The plant contains yellow juice, and the vegetative parts, particularly the stem and leaves, have a bluish-green leathery texture. The morphology of *A. mexicana* described in this study is consistent with findings from research conducted in Yemen [36] and the Chihuahuan Desert regions in Mexico [37]. Interestingly, this study revealed two distinguishable stomatal patterns: anisocytic and actinocytic. These results provide additional depth to prior research conducted in Yemen and Mali, which primarily found that actinocytic stomata and emphasized the absence of trichomes [36; 38]. The dual stomatal patterns observed in this study may indicate adaptive traits for regulating water utilization in diverse environmental conditions, a crucial attribute for a plant flourishing in semi-arid and desert climates. Additionally, the flowers observed in this study, with 4–6 bright yellow petals and a spherical bud shape, mirror morphological traits mentioned in several studies [39; 40], where flower dimensions and coloration have an important role in pollination biology. The results on seed morphology -brown and spherical seeds with reticulated surfaces align with the Chihuahuan Desert study [37], which described similar seed morphologies adapted for spreading in arid regions. These comparative observations may confirm the adaptability of *A. mexicana* to various ecological environments, including arid and semi-arid habitats, suggesting its resilience and evolutionary acclimations.

### 2. Allelopathic effects

The present study indicated that the allelopathic effects of *A. mexicana* leaf and seed aqueous extracts inhibited the germination of both crops *T. aestivum* and *H. vulgare*. This result is very common to many weed extracts used to treat crop seed germination [41–43]. Our finding aligns with those of Watakhere et al. (2023) [44] indicating that *A. Mexicana* extracts have a concentration dependent effect on wheat germination and seedling length, potentially due to the presence of allelochemicals that induce changes in physiological and biochemical processes necessary for seed germination [45]. Such allelochemicals showed the ability to suppress *Phaseolus vulgaris* and *Zea mays* seed germination and growth even at low concentrations of *A. mexicana* extracts (Ojija, 2023) [46]. Similarly, it was established that *A. mexicana* extracts reduced *Solanum lycopersicum* germination and seedling length López et al. (2023) [47]. The allelochemicals present in *A. mexicana* can cause disruption of mitochondrial respiration and disrupt the activity of metabolic enzymes that participate in glycolysis [48].

In the current study, *A. mexicana* extracts also affected the other germination variables, including the germination speed and the mean daily germination. Similarly, Ojija, 2023 [47] have found a phytotoxic effect of the moss *Thuidium Kanedae* on germination percentage, germination index and germination potential of the flowering plant *Taraxacum mongolicum*. Mlombo et al. (2024) [42] have indicated that extracts obtained from *Argemone ochroleuca* delayed germination percentage, mean germination time, germination speed, germination index and mean daily germination of soybean. The observed delay in seed germination can have some important biological and ecological consequences, since it affects the ability of the seedling to establish itself in natural conditions, resulting in uneven plant stand [49]. Our results indicated that at high extract concentrations, the different plant parts of *A. mexicana* have revealed different allelopathic effects on the germination of *T. aestivum* and *H. vulgare* (Tables 2 and 3). These findings align with those of Namkeleja et al. (2013) [50] showing that *A. mexixana* seed extracts had more inhibitory effect on *Brachiaria dictyoneura* germination compared to extracts obtained from the leaves. Paul and Begum (2007) [51] have explained the different inhibitory potential of *A. mexicana* plant parts extracts on varying organs responsible for synthesis and storage of allelopathic phytochemicals.

Previous work of Namkeleja et al. (2014) [52] agreed with the here described high inhibitory effect of *A. mexicana* seeds and showed its effect on the soil toxification. Such a finding can implicate to the research of good practices for soil rehabilitation such as growing cover crops after *A. mexicana* invasion. Farmers can also use soil flushing to minimize the allelopathic compound concentrations in the soil.

Allelopathy has been studied in numerous plants and has demonstrated a sub-stantial impact on the growth and physiology of plant seedlings. Li et al. (2024) [53] found that the allelopathic effect of rice straw in suppressing seedling growth in wheat. On the other hand, Deng et al. (2024) [54] have found that a leaf aqueous solution of *Eucalyptus robusta* Sm. enhances the elongation of rapeseed radicle. In the present investigation and throughout the nine days of treatment, the radicles exhibited sensitivity to low concentration of *A. mexicana* leaf and seed extracts for both examined species *T. aestivum* and *H. vulgare*. For instance, at the lowest concentration (5%), both extracts significantly impaired radicle elongation. This result is in alignment with the findings of Samal et al. (2023) [55] who noted the suppression of root elongation of *Vigna Mungo* caused by *A. mexicana* leaf and seed extracts. Similarly, invasive *A. mexicana* has been claimed to inhibit the root growth of 4 varieties of *Vigna mungo* L., 3 varieties of *Brassica campestris* L. and 4 varieties of *Triticum aestivum* L. [52]. The phenomenon of reducing root system in response to invasive species was previously observed in other plants such *as Juglans regia* L. and *Solidago canadensis* L. and in the crop species; *Brassica oleracea*, *Fagopyrum esculen-tum*, *Lupinus albus*, and *Triticum aestivum* [56]. More recent evidence [57] suggests that the allelochemicals found in the aqueous extract of invasive plants can modify cellular membrane integrity, inhibit apical cell division, and reduce the growth of embryonic roots and axes. These modifications collectively could impede the root system elongation and therefore lead to a reduction in radicle length.

The findings of the current study show the significant reduction of the plumule elongation of both studied species *T. aestivum* and *H. vulgare* that subjected to *A. mexicana* leaf and seed extracts. These findings correspond with those of Siddiqui et al. (2002) [58] who showed the inhibitory effect of *A. mexicana* extract on the growth of tomato shoot. In comparative analyses between the control treatment and the aqueous extracts treatment, it has been noted that the root lengths were more significantly affected than shoot lengths. Similar observation was established by Sarkar et al. (2012) [59] who have studied the potential allelopathic effect of *Cassia tora* on the seed germination and growth of *Brassica campestris* L. and have found the suppressive impact of allelochemicals exhibited more on the roots comparing to the shoots. The differential inhibitory impact of *A. mexicana* extracts on roots and shoots was attributed to the roots’ interaction with the filter paper, resulting in a constant absorption of the extract solution [59; 60] in addition to the higher root permeability and sensitivity toward allelochemicals comparing to shoots [61; 62]. Moreover, Chon et al. (2000) [62] have postulated that root length is a good indicator of allelopathic effect of plant extracts, as it exhibits greater sensitivity to phytotoxic compounds than shoot growth. Root and shoot lengths are critical parameters that determine plants’ growth and health, as they are essential for nutrient uptak and physical support of the plant. Thus, the inhibitory effect of invasive plants on root and shoot elongation may adversely affect crop production.

In the present work, it has been shown that *H. vulgare* exhibited lower sensitivity to *A. mexicana* leaf extract in comparison to *T. aestivum*. Similar to this study, Grul’ová et al. (2024) [63] demonstrated that the allelopathic effects of *Heracleum mantegazzianum* extract were more significant on *T. aestivum* than on *H. vulgare*. The here investigated difference in sensitivity to *A. mexicana* can be beneficial for agricultural management, *e.g.,* the cultivation of *H. vulgare* rather than *T. aestivum* in the area prone to the invasive species to reduce yield loss. What’s more, the culture of the less sensitive crop would reduce herbicide use.

Investigations of Burhan and Shaukat (1999) [64] on the germination and growth of *T. aestivum* treated with the shoot aqueous extract obtained from *A. mexicana* revealed the inhibitory effect of this extract on the *T. aestivum* and were attributable to the presence of the phenolic compounds including p-hydroxybenzoic acid, vanillic acid and salicylic acid. The experimental work of Huang et al. (2020) [65] found that the growth of *Cucumis sativus* L. seedlings was reduced when treated with high concentrations of phy-droxybenzoic acid. In addition, Ma et al. 2023 [66] have postulated that vanillin posed allelopathic effect that inhibited the growth of *Solanum tuberosum* L. when applied a recent study discovered that cinnamic acid treatment decreased root elongation in *Cucumis sativus* L. [67].

*A. mexicana* is increasingly encroaching upon agricultural lands [45]. Its potential to suppress germination and early–growth [46] caused marked declines in crop yields thereby threatening food security and the livelihoods of farmers [68; 69]. On the other hand, it was found that exploring allelopathic plants like *A. mexicana* offers a great implication in the potential strategies for the development of ecologically friendly bioherbicides. Considering the environmental impacts caused by the use of the chemical control method and the growing number of species resistant to the different mechanisms of action of herbicides [70] (Miller, 2024), the study of allelopathy is promising for sustainable agriculture. However, it is important to emphasize the need for further studies to verify the efficiency of the extracts in the emergence of seeds under field conditions and, later, to evaluate the possibility of using these species as raw material for the development of formulations to be inserted in the management of weeds.

## VI. Conclusion

This study demonstrated that aqueous extracts derived from the seeds and leaves of *Argemone mexicana* exert significant inhibitory effects on the germination and early growth of *Triticum aestivum* and *Hordeum vulgare*. The degree of inhibition increased proportionally with extract concentration, as evidenced by the progressive decline in germination rates, plumule and radicle elongation, and dry biomass accumulation. These findings confirm the allelopathic potential of *A. mexicana* and highlight its capacity to interfere with the early developmental stages of economically important cereal crops.

To deepen our understanding of the underlying mechanisms, further research is warranted to isolate and characterize the specific allelochemicals involved, and to elucidate their modes of action at physiological and molecular levels. Such investigations are essential not only to clarify the biochemical pathways responsible for the observed phytotoxicity, but also to assess species-specific responses among crops and weeds. This knowledge could inform the development of novel, plant-based strategies for sustainable weed management, particularly in agroecosystems where *A. mexicana* is prevalent.

From a practical standpoint, the results suggest that the proximity of *A. mexicana* to cultivated fields may pose a risk to wheat and barley production. It is therefore advisable to avoid sowing these crops in areas where *A. mexicana* is established or likely to proliferate, as part of an integrated crop management approach aimed at minimizing allelopathic interference and optimizing yield performance.
